# Urinary dipstick findings and UTI risk with SGLT2 inhibitors: a post-hoc analysis of the CANVAS and CREDENCE trials

**DOI:** 10.1093/ndt/gfag062

**Published:** 2026-03-16

**Authors:** Yohei Doi, Takayuki Hamano, Mayumi Fukuda-Doi, Yoshitaka Isaka

**Affiliations:** Department of Nephrology, Graduate School of Medicine, The University of Osaka, Suita, Osaka, Japan; Department of Cardiology, Pulmonology, Hypertension & Nephrology, Ehime University Graduate School of Medicine, Toon, Ehime, Japan; Department of Nephrology, Graduate School of Medicine, The University of Osaka, Suita, Osaka, Japan; Department of Nephrology, Nagoya City University Graduate School of Medical Sciences, Nagoya, Aichi, Japan; Department of Data Science, National Cerebral and Cardiovascular Center, Suita, Osaka, Japan; Department of Nephrology, Graduate School of Medicine, The University of Osaka, Suita, Osaka, Japan

**Keywords:** SGLT2 inhibitor, type 2 diabetes mellitus, urinary dipstick test, urinary tract infection

## Abstract

**Background:**

Despite established cardiorenal benefits, concerns about urinary tract infection (UTI) risk may limit sodium-glucose cotransporter 2 inhibitor (SGLT2i) use, especially in high-risk patients. We hypothesized that urine leukocyte esterase (LE) and nitrite—findings compatible with pyuria or bacteriuria—predict subsequent UTI risk and modify the association between canagliflozin and UTIs.

**Methods:**

We conducted a post-hoc analysis of individual-participant data from two randomized, double-blind, placebo-controlled trials of canagliflozin in type 2 diabetes. The primary outcome was time to first UTI; the secondary outcome was total UTI events, analyzed with Cox and Andersen–Gill models.

**Results:**

Among 8614 participants, abnormal LE and nitrite were observed in 10.7% and 3.5%, respectively. After adjustment, the risk of first UTI increased with greater LE positivity [hazard ratio (HR) 1.72, 1.92, 2.81 for 1+, 2+, 3+ vs normal]. Abnormal nitrite was also associated with higher risk [HR 2.28 (1+), 1.66 (2+)]. Similar findings were observed for total UTI events. Overall, canagliflozin did not increase risk of first [HR 1.07; 95% confidence interval (CI) 0.93–1.22] or total UTI events (HR 1.01; 95% CI 0.86–1.17). LE and nitrite results did not significantly modify the effect of canagliflozin on the primary outcome. For LE, the HR for first UTI was 1.17 (95% CI 1.00–1.38) in the normal group and 0.94 (0.73–1.21) in the abnormal group (*P*_interaction_ = .10). For nitrite, the HRs were 1.08 (0.94–1.24) and 0.92 (0.59–1.43), respectively (*P*_interaction_ = .45). For the secondary outcome, canagliflozin also did not increase the risk of total UTI events in either the abnormal LE or nitrite group [HR 0.76 (0.58–0.99) and 1.18 (0.79–1.76), respectively].

**Conclusions:**

Although abnormal LE and nitrite were associated with increased UTI risk, canagliflozin did not further increase this risk in these subgroups. These findings do not support routine withholding of SGLT2i in patients with dipstick findings suggestive of pyuria or bacteriuria.

## Graphical Abstract

**Graphical Abstract:**
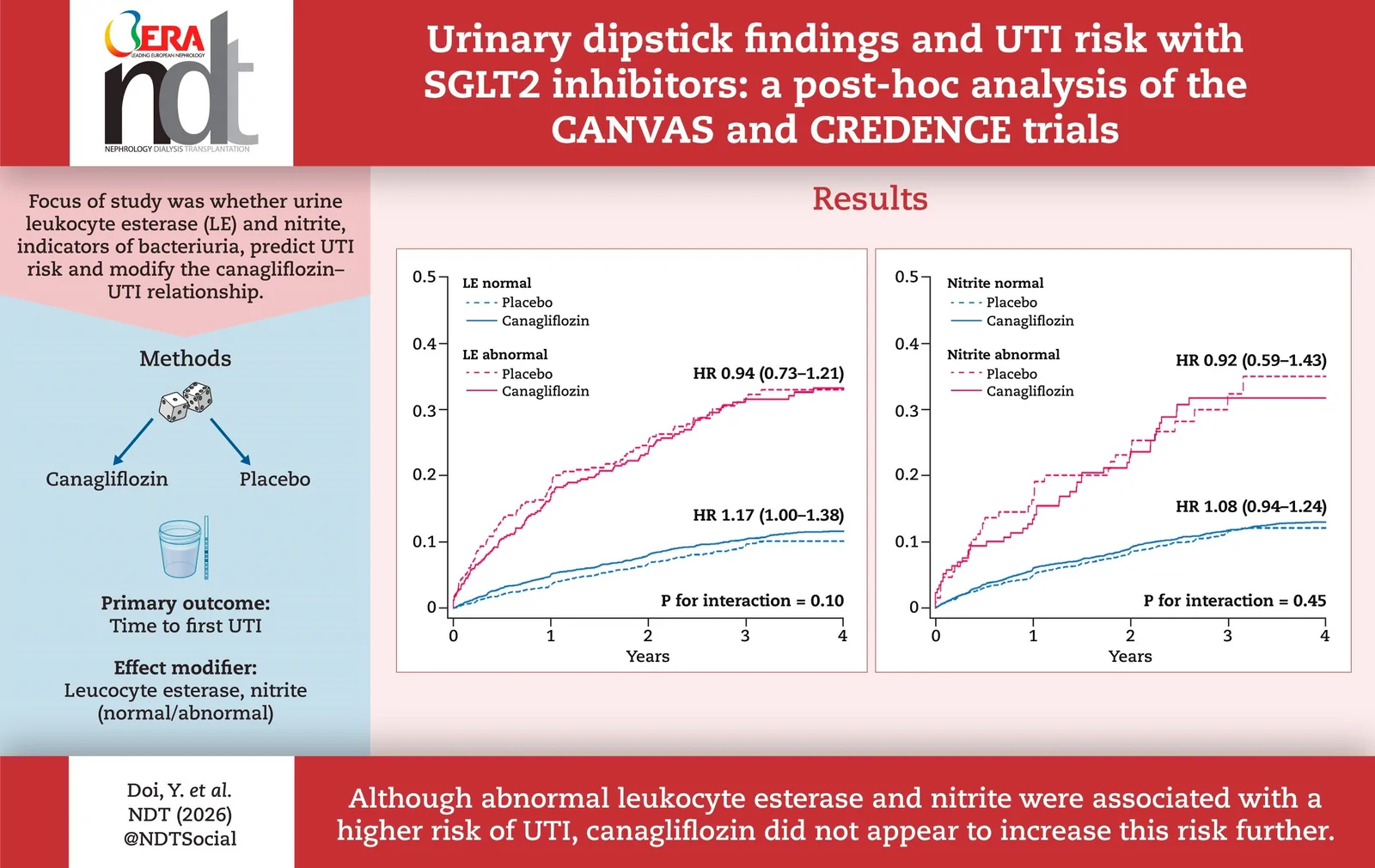

KEY LEARNING POINTS **What was known:**

- Urinary tract infections (UTIs) are common and burdensome in patients with type 2 diabetes mellitus.
- Simple and widely available biomarkers for predicting UTI risk are scarce.
- Concerns about sodium-glucose cotransporter 2 inhibitor (SGLT2i)-associated UTIs can hinder their use, especially in high-risk patients, potentially impeding the delivery of optimal care.

**This study adds:**

- Abnormal leukocyte esterase and nitrite results were associated with a significantly higher risk of subsequent UTIs, irrespective of canagliflozin use.
- Canagliflozin did not increase UTI risk, even in patients with abnormal dipstick findings.

**Potential impact:**

- Dipstick-based risk stratification may help guide simple, targeted UTI prevention strategies—especially in primary care and resource-limited settings.
- These findings may help support guideline-directed use of SGLT2i and inform decisions to avoid unnecessary withholding in patients with dipstick results compatible with pyuria or bacteriuria.

## INTRODUCTION

Urinary tract infection (UTI) is a prevalent condition with substantial clinical and economic consequences, particularly among patients with type 2 diabetes mellitus (T2DM) [1–3]. While asymptomatic bacteriuria (ASB) has been reported as a biomarker for predicting UTI development in this population [4–7], its routine use is limited by the need for urine culture, which is time-consuming and resource-intensive. In contrast, dipstick tests are inexpensive, rapid and widely utilized for kidney disease screening [8]. Despite their broad application, key dipstick parameters—leukocyte esterase (LE) and nitrite (NIT), key diagnostic markers for UTIs—are overlooked in asymptomatic individuals. The predictive value of LE and NIT for future UTI risk remains uncertain. If LE and/or NIT can be shown to predict UTI risk, particularly in asymptomatic patients with T2DM, this would enhance the clinical value of dipstick testing and inform preventive strategies.

Sodium-glucose cotransporter 2 inhibitors (SGLT2i) are pivotal in the management of T2DM, heart failure and chronic kidney disease, offering significant renal and cardiovascular protective benefits [9–11]. However, their mechanism of action, which increases urinary glucose excretion, raises concerns about higher UTI risk [12]. Previous studies have yielded mixed results regarding whether SGLT2i increase UTI incidence [13–15], highlighting the need to define this association in high-risk patients. Determining whether SGLT2i increase UTI risk in such populations is critical for clinical decision-making and optimizing patient care.

To address these gaps, this study leverages datasets from the CANagliflozin cardioVascular Assessment Study (CANVAS) [16] and the Canagliflozin and Renal Events in Diabetes with Established Nephropathy Clinical Evaluation (CREDENCE) [17] trials—both double-blind, placebo-controlled evaluations of canagliflozin in patients with T2DM—to [1] assess whether dipstick LE and NIT predict UTI risk and [2] determine whether these parameters modify the effect of canagliflozin on UTI outcomes.

## MATERIALS AND METHODS

### Data sharing

Clinical data from CANVAS and CREDENCE are publicly accessible through the Yale University Open Data Access Project (http://yoda.yale.edu/). Ethical approval was obtained from committees at each site (ClinicalTrials.gov: NCT01032629, NCT02065791), adhering to the Declaration of Helsinki. All participants provided written informed consent.

### Study design and participants

This study is a post-hoc pooled analysis using individual participant data (IPD) from the CANVAS and CREDENCE trials. Details of the study design, interventions and primary outcomes have been published previously [16, 17]. Both were randomized, double-blind, placebo-controlled, multicenter trials evaluating canagliflozin in patients with T2DM. In CANVAS, participants were randomized (1:1:1) to receive canagliflozin 300 mg, canagliflozin 100 mg or placebo. In CREDENCE, randomization was (1:1) to receive canagliflozin 100 mg or placebo. CANVAS enrolled patients with T2DM and HbA1c 7.0%–10.5%, aged ≥30 years with atherosclerotic vascular disease, or ≥50 years with at least two cardiovascular risk factors. CREDENCE enrolled patients with T2DM and HbA1c 6.5%–12.0%, aged ≥30 years, with an estimated glomerular filtration rate (eGFR) of 30–<90 mL/min/1.73 m² and a urinary albumin-to-creatinine ratio (UACR) of 300–5000 mg/g while receiving renin–angiotensin system blockade. Data from the CANVAS-R trial (ClinicalTrials.gov identifier: NCT01989754) were excluded because only serious adverse events or those leading to drug discontinuation were collected.

### The measurements of urine leukocyte esterase and nitrite test

Urine dipstick testing was performed at baseline and every 52 weeks thereafter. In the CANVAS trial, an additional test was conducted at Week 18. All analyses were performed in a central laboratory. LE results of “negative” or “trace” were classified as normal, and “1+,” “2+” and “3+” as abnormal. Similarly, NIT results of “negative” were considered normal, while “1+” and “2+” were classified as abnormal.

### Outcomes

The primary outcome was the first UTI event, and the secondary outcome was total (first and recurrent) UTI events. The exploratory outcome was the incidence of mycotic genital infections (MGIs). Outcomes were recorded as investigator-reported adverse events and defined according to pre-specified MedDRA Preferred Terms in the CANVAS and CREDENCE trials [16, 17]. In CANVAS, UTI data after 7 January 2014 were excluded because only serious adverse events or those leading to discontinuation were recorded thereafter. In contrast, MGIs were recorded throughout the entire trial period regardless of severity. Accordingly, MGI data from the full study period were used, whereas UTI data were limited to events collected up to 7 January 2014 (Supplementary data, Table S1).

### Statistical analysis

Analyses included participants with available baseline dipstick results, based on the on-treatment population defined *a priori* as patients within 30 days of their last trial dose. Baseline characteristics were summarized as mean ± SD or median [interquartile range (IQR)] for continuous variables and as percentages for categorical variables. Kaplan–Meier curves and Cox regression were used to examine the association between LE/NIT categories and time to first event. Three models were applied: Model 1 (unadjusted); Model 2 [adjusted for age, sex and treatment group (canagliflozin vs placebo)]; and Model 3 (fully adjusted for race, cerebrovascular disease, diabetes duration, body mass index, HbA1c, eGFR, UACR and the use of diuretics and insulin). These variables were selected based on clinical relevance and data availability. Total UTI events were analyzed using the Andersen–Gill model. The effects of canagliflozin (pooled doses) versus placebo on outcomes were evaluated with Cox and Andersen–Gill models adjusted for age and sex. Interaction terms were added to assess effect modification by baseline LE/NIT categories. The proportional hazards assumption was evaluated visually using log–cumulative hazard plots, and time-varying effects were illustrated with the Stata command “stphcoxrcs” [18]. Where violations were identified, time-varying coefficients or accelerated failure time models were used. Mixed-effects logistic regression was used to examine the effect of canagliflozin versus placebo on longitudinal binary LE/NIT outcomes, including fixed effects for treatment, age, sex, visit and baseline LE/NIT category, and random intercepts for within-individual variability. All tests were two-sided, with *P* < .05 considered significant. Given no adjustment for multiplicity, results should be considered hypothesis-generating. Analyses were performed using Stata/SE 18 (StataCorp, College Station, TX, USA).

## RESULTS

### Baseline characteristics

Baseline dipstick data were available for 98.7% (8614/8724) of the on-treatment population (Supplementary data, Fig. S1). Of these, 34.8% (2915/8614) were female, the mean age was 62 years and the median follow-up was 2.8 years (IQR 1.9–4.1) for the on-treatment UTI analysis and 3.5 years (IQR 2.5–6.1) for the MGI analysis (Table 1, Supplementary data, Table S1). For LE, 89.3% (7693/8614) were normal, while 1+, 2+ and 3+ were observed in 3.3% (285/8614), 2.8% (243/8614) and 4.6% (393/8614), respectively. For NIT, 96.5% (8312/8614) were normal, with 1+ in 1.1% (91/8614) and 2+ in 2.5% (211/8614). Concurrent abnormalities in both LE and NIT were present in 2.0% (169/8614) (Supplementary data,Fig. S2). Patients with abnormal LE results were generally older, more likely to be female, and had longer diabetes duration, lower eGFR and higher UACR (Table 1, Supplementary data, Fig. S3). Patients with abnormal NIT results were also older and more frequently female, with higher HbA1c levels. These patterns were consistent in both sexes (Supplementary data, Tables S2 and S3).

**Table 1:** Baseline characteristics of the study population, stratified by LE and NIT test results.^a^

	LE test				NIT test		
	Normal	(1+)	(2+)	(3+)	Normal	(1+)	(2+)
Characteristics	*N* = 7693	*N* = 285	*N* = 243	*N* = 393	*N* = 8312	*N* = 91	*N* = 211
Age, years	62 (9)	63 (8)	64 (9)	64 (9)	62 (9)	62 (8)	64 (8)
Female sex, *n* (%)	2231 (29.0)	222 (77.9)	189 (77.8)	273 (69.5)	2672 (32.1)	70 (76.9)	173 (82.0)
Race, *n* (%)^b^							
White	5385 (70.0)	203 (71.2)	162 (66.7)	265 (67.4)	5807 (69.9)	67 (73.6)	141 (66.8)
Black or African American	282 (3.7)	17 (6.0)	13 (5.3)	16 (4.1)	317 (3.8)	4 (4.4)	7 (3.3)
Asian	1485 (19.3)	50 (17.5)	46 (18.9)	74 (18.8)	1591 (19.1)	14 (15.4)	50 (23.7)
Other	541 (7.0)	15 (5.3)	22 (9.1)	38 (9.7)	597 (7.2)	6 (6.6)	13 (6.2)
Current smoker, *n* (%)	1289 (16.8)	35 (12.3)	26 (10.7)	40 (10.2)	1353 (16.3)	8 (8.8)	29 (13.7)
History of hypertension, *n* (%)	7074 (92.0)	272 (95.4)	232 (95.5)	368 (93.6)	7663 (92.2)	88 (96.7)	195 (92.4)
History of CV disease, *n* (%)	4247 (55.2)	129 (45.3)	118 (48.6)	206 (52.4)	4556 (54.8)	48 (52.7)	96 (45.5)
History of cerebrovascular disease, *n* (%)	1227 (15.9)	42 (14.7)	46 (18.9)	72 (18.3)	1336 (16.1)	16 (17.6)	35 (16.6)
Diabetes duration, years	14.5 (8.2)	15.0 (8.0)	14.7 (8.0)	15.9 (8.6)	14.6 (8.2)	14.6 (8.0)	14.2 (8.2)
Body mass index, kg/m^2^ ^c^	32 (6)	32 (6)	32 (6)	32 (7)	32 (6)	32 (7)	33 (7)
SBP, mmHg	138 (16)	139 (16)	140 (16)	138 (17)	138 (16)	140 (17)	139 (16)
DBP, mmHg	78 (10)	77 (10)	78 (9)	77 (10)	78 (10)	78 (11)	77 (9)
Glycated hemoglobin, %	8.2 (1.1)	8.3 (1.1)	8.3 (1.2)	8.3 (1.2)	8.2 (1.1)	8.5 (1.2)	8.3 (1.2)
eGFR, mL/min/1.73 m^2^	67 (21)	62 (21)	63 (22)	60 (20)	67 (21)	66 (20)	66 (21)
UACR, mg/g	243 (12–974)	241 (17–1118)	250 (17–1061)	391 (29–1190)	255 (12–995)	293 (27–1219)	117 (18–731)
Total cholesterol, mmol/L	4.49 (1.23)	4.82 (1.35)	4.70 (1.20)	4.55 (1.28)	4.50 (1.23)	4.86 (1.60)	4.73 (1.35)
HDL cholesterol, mmol/L	1.16 (0.33)	1.23 (0.33)	1.25 (0.36)	1.21 (0.35)	1.17 (0.33)	1.16 (0.32)	1.28 (0.37)
LDL cholesterol, mmol/L	2.38 (1.00)	2.57 (1.06)	2.52 (0.99)	2.44 (1.07)	2.39 (1.00)	2.57 (1.15)	2.60 (1.14)
Triglycerides, mmol/L	1.74 (1.25–2.48)	1.76 (1.29–2.55)	1.74 (1.32–2.54)	1.73 (1.29–2.36)	1.74 (1.26–2.48)	1.75 (1.24–2.59)	1.61 (1.23–2.29)
Medication, *n* (%)							
ACEi/ARB	6939 (90.2)	260 (91.2)	229 (94.2)	364 (92.6)	7509 (90.3)	86 (94.5)	197 (93.4)
Diuretics	3460 (45.0)	142 (49.8)	119 (49.0)	185 (47.1)	3758 (45.2)	44 (48.4)	104 (49.3)
Statin	5479 (71.2)	197 (69.1)	160 (65.8)	257 (65.4)	5900 (71.0)	55 (60.4)	138 (65.4)
Insulin	4432 (57.6)	179 (62.8)	147 (60.5)	240 (61.1)	4817 (58.0)	52 (57.1)	129 (61.1)

a Data are presented as mean (standard deviation) or median (IQR) for continuous measures, and *n* (%) for categorical measures.

^b^Race was reported by the patients. Other includes American Indian or Alaska Native, Native Hawaiian or other Pacific Islander, multiple, other, unknown and not reported.

^c^The body mass index is the weight in kilograms divided by the square of the height in meters. CV, cardiovascular; SBP, systolic blood pressure; DBP, diastolic blood pressure; eGFR, estimated glomerular filtration rate; HDL, high-density lipoprotein; LDL, low-density lipoprotein; ACEi, angiotensin-converting enzyme inhibitors; ARB, angiotensin receptor blockers.

### Prediction of urinary tract infections using leukocyte esterase and nitrite test

Kaplan–Meier curves demonstrated significant differences in UTI incidence across baseline LE and NIT categories (both log-rank *P* < .01; Fig. 1). For LE, individuals with normal results (reference group) had the lowest UTI risk, with a stepwise increase across 1+ to 3+ categories [hazard ratio (HR) and 95% confidence interval (CI) in Model 1: 2.96 (2.29–3.82), 3.21 (2.24–4.17) and 4.52 (3.73–5.49), respectively; Table 1]. This association remained robust after adjustment for clinically relevant covariates. Similar patterns were observed for total UTI events. For NIT, abnormal results were also associated with higher UTI risk compared with normal results, with HRs of 3.60 (2.47–5.25) for 1+ and 2.78 (2.09–3.69) for 2+. This association was evident for both first and total UTI, although a clear dose–response gradient was not observed (Table 2, Fig. 1). Sex-based effect modification was observed for LE (*P*_interaction_ < .01) in the primary outcome, with a stronger association between abnormal LE and UTI risk in males than in females. In contrast, NIT showed no evidence of effect modification by sex (*P*_interaction_ = .30; Supplementary data, Table S4). Similar sex-based effect modification for LE was also present for total UTI events, whereas no sex interaction was detected for NIT in this outcome (data not shown).

**Figure 1:**
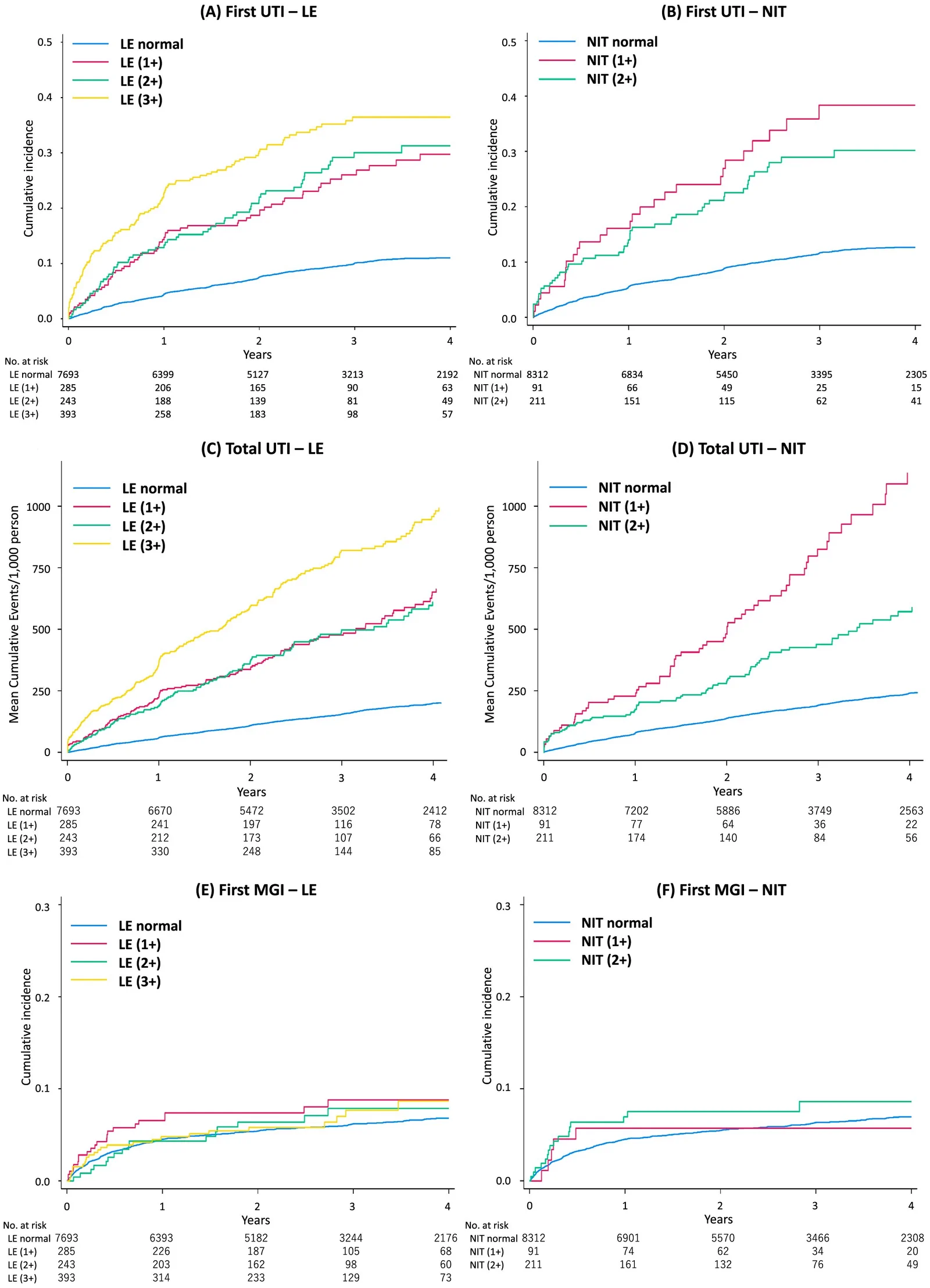
Risk of UTI and MGI by baseline dipstick results. (**A, B**) Kaplan–Meier curves for time to first UTI by LE and NIT categories. (**C, D**) Cumulative incidence plots for total UTI events by LE and NIT categories. (**E, F**) Kaplan–Meier curves for time to first MGI by LE and NIT categories.

**Table 2:** Associations between baseline urine LE and NIT test results and risk of UTIs and MGIs.

			Model 1 (Crude)		Model 2^a^		Model 3^b^	
	Events, *n*/*N* (%)	Event rate (per 1000 PY)	HR (95% CI)	*P* for trend	HR (95% CI)	*P* for trend	HR (95% CI)	*P* for trend
First UTI								
Whole	903/8614 (10.5)	41.7						
LE test								
Normal	657/7693 (8.5)	33.3	ref	<0.01	ref	<0.01	ref	<0.01
(1+)	64/285 (22.5)	101.7	2.96 (2.29–3.82)		1.78 (1.37–2.32)		1.72 (1.32–2.24)	
(2+)	61/243 (25.1)	110.5	3.21 (2.47–4.18)		1.95 (1.50–2.56)		1.92 (1.47–2.52)	
(3+)	121/393 (30.8)	160.3	4.52 (3.73–5.49)		2.91 (2.38–3.55)		2.81 (2.30–3.44)	
NIT test								
Normal	824/8312 (9.9)	39.2	ref	<0.01	ref	<0.01	ref	<0.01
(1+)	28/91 (30.8)	149.2	3.61 (2.47–5.25)		2.25 (1.54–3.29)		2.28 (1.56–3.34)	
(2+)	51/211 (24.2)	113.2	2.78 (2.10–3.69)		1.62 (1.21–2.15)		1.66 (1.25–2.21)	
Total (first and recurrent) UTIs								
Whole	1561/8614	67.5						
LE test								
Normal	1070/7693	51.5	ref	<0.01	ref	<0.01	ref	<0.01
(1+)	125/285	169.2	3.27 (2.52–4.24)		1.93 (1.45–2.57)		1.89 (1.43–2.52)	
(2+)	105/243	162.5	3.15 (2.39–4.14)		1.87 (1.41–2.48)		1.83 (1.39–2.42)	
(3+)	261/393	270.3	5.18 (4.17–6.43)		3.25 (2.59–4.07)		3.14 (2.51–3.92)	
NIT test								
Normal	1417/8312	63.3	ref	<0.01	ref	<0.01	ref	<0.01
(1+)	63/91	270.8	4.24 (2.85–6.30)		2.50 (1.72–3.65)		2.52 (1.73–3.68)	
(2+)	81/211	150.7	2.37 (1.83–3.06)		1.33 (1.02–1.72)		1.36 (1.05–1.76)	
First MGI								
Whole	524/8614 (6.1)	20.9						
LE test								
Normal	461/7693 (6.0)	20.2	ref	0.27	ref	0.64	ref	0.09
(1+)	22/285 (7.7)	30.9	1.38 (0.90–2.12)		1.24 (0.80–1.91)		1.37 (0.88–2.15)	
(2+)	16/243 (6.6)	25.0	1.14 (0.69–1.87)		1.01 (0.61–1.67)		1.12 (0.68–1.87)	
(3+)	25/393 (6.4)	26.5	1.15 (0.77–1.72)		1.08 (0.71–1.62)		1.37 (0.91–2.08)	
NIT test								
Normal	824/8312 (9.9)	20.6	ref	0.26	ref	0.70	ref	0.47
(1+)	5/91 (5.5)	21.7	0.96 (0.40–2.32)		0.78 (0.32–1.90)		0.91 (0.37–2.20)	
(2+)	16/211 (7.6)	30.8	1.36 (0.83–2.24)		1.16 (0.70–1.92)		1.24 (0.75–2.06)	

a Model 2: adjusted for age, sex, treatment (canagliflozin vs placebo).

b Model 3: adjusted for age, sex, treatment (canagliflozin vs placebo), race, history of cerebrovascular disease, diabetes duration, body mass index, glycated hemoglobin, eGFR, UACR, diuretic use and insulin use. PY, person-years; eGFR, estimated glomerular filtration rate.

### The independent significance of leukocyte esterase and nitrite in predicting urinary tract infections

When LE and NIT were included simultaneously in Model 3, both were independently associated with first UTI events (LE: HR 2.09; 95% CI 1.78–2.47; NIT: HR 1.41; 95% CI 1.11–1.80). For total UTI events, LE remained independently associated (HR 2.30; 95% CI 1.90–2.79), whereas the association for NIT was attenuated (HR 1.27; 95% CI 0.99–1.63). No interaction between LE and NIT was observed for either outcome (*P*_interaction_ = .24 and .69).

### The impact of canagliflozin on urinary tract infections and leukocyte esterase and nitrite test results

Canagliflozin did not significantly affect the risk of first UTI events in the overall population (HR 1.07; 95% CI 0.93–1.22; Table 3, Fig. 2). For total UTI events, the incidence and the distribution of recurrent episodes were also similar between treatment groups (HR 1.01; 95% CI 0.86–1.17; Figs 2 and 3). UTI events leading to drug withdrawal were also comparable between the placebo and canagliflozin group [2.1% (13/619) vs 2.9% (27/942), respectively]. Mixed-effects logistic regression also showed no significant effect of canagliflozin on longitudinal LE or NIT results [odds ratio (OR) 1.02; 95% CI 0.84–1.24, and OR 0.87; 95% CI 0.66–1.14, respectively; Supplementary data, Fig. S4).

**Figure 2:**
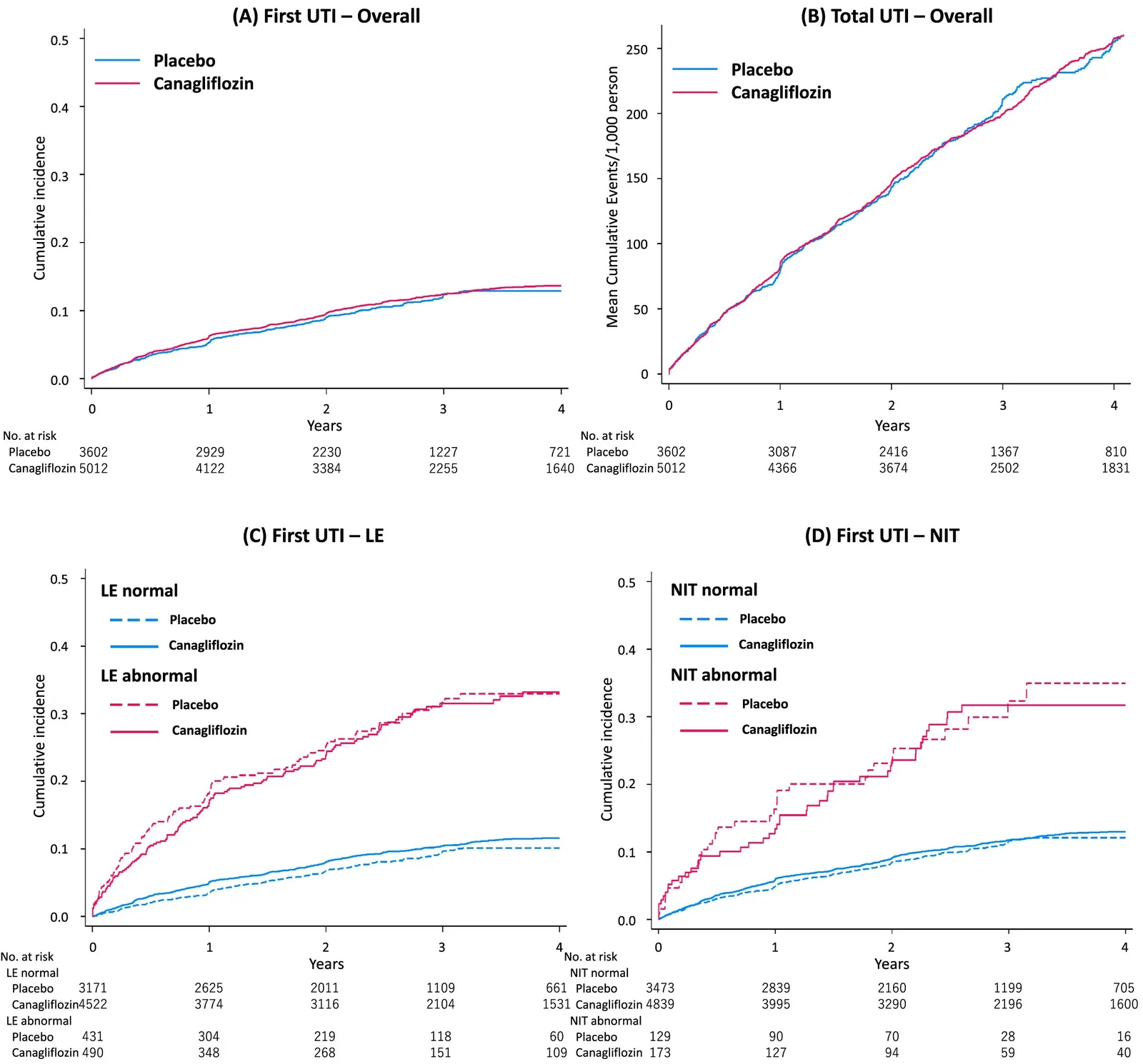

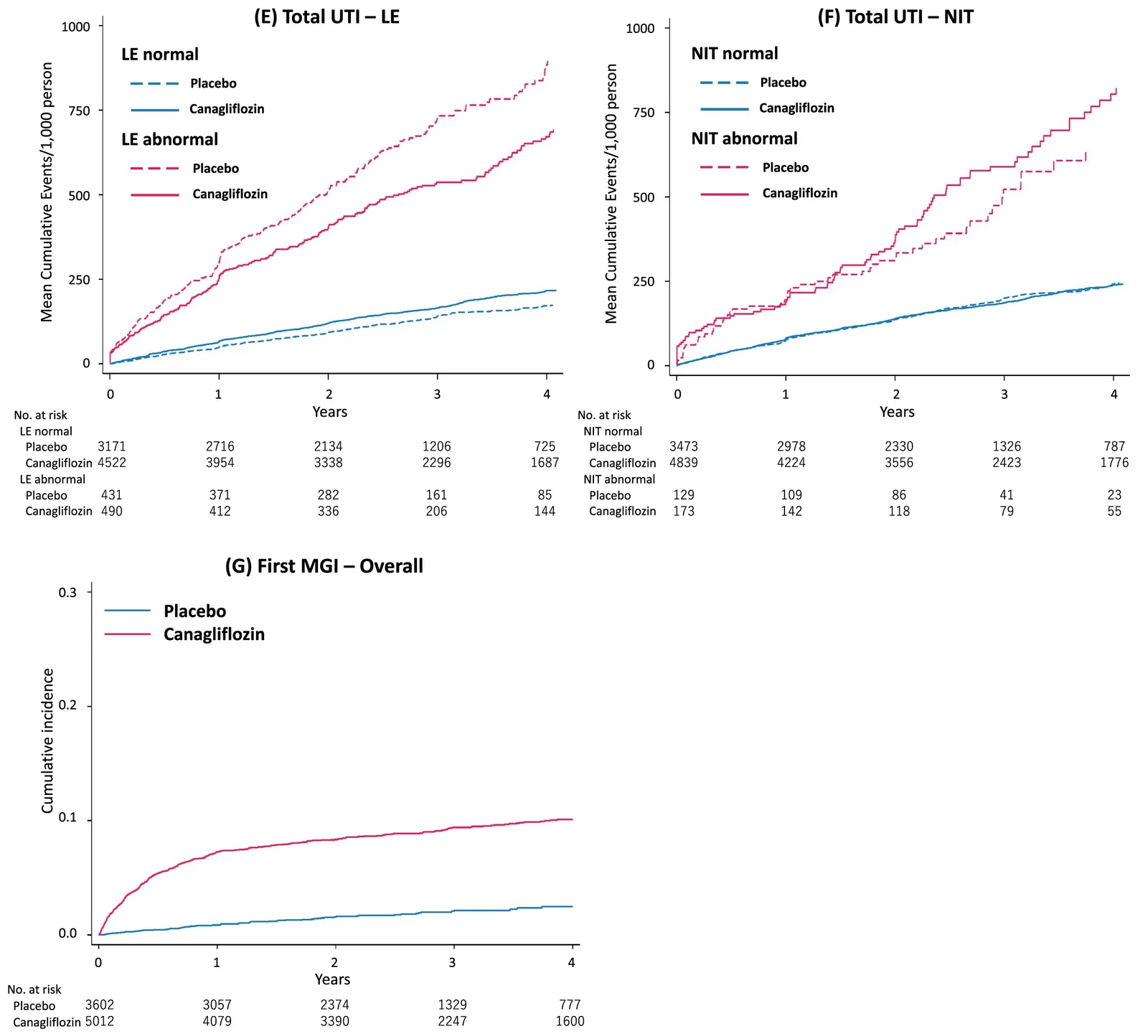
Effect of canagliflozin on UTI and MGI. (**A, B**) UTI risk (time to first event and total events) by treatment group (canagliflozin vs placebo). (**C**–**F**) UTI outcomes by baseline LE and NIT, stratified by treatment group. (**G**) Time to first MGI by treatment group.

**Figure 3:**
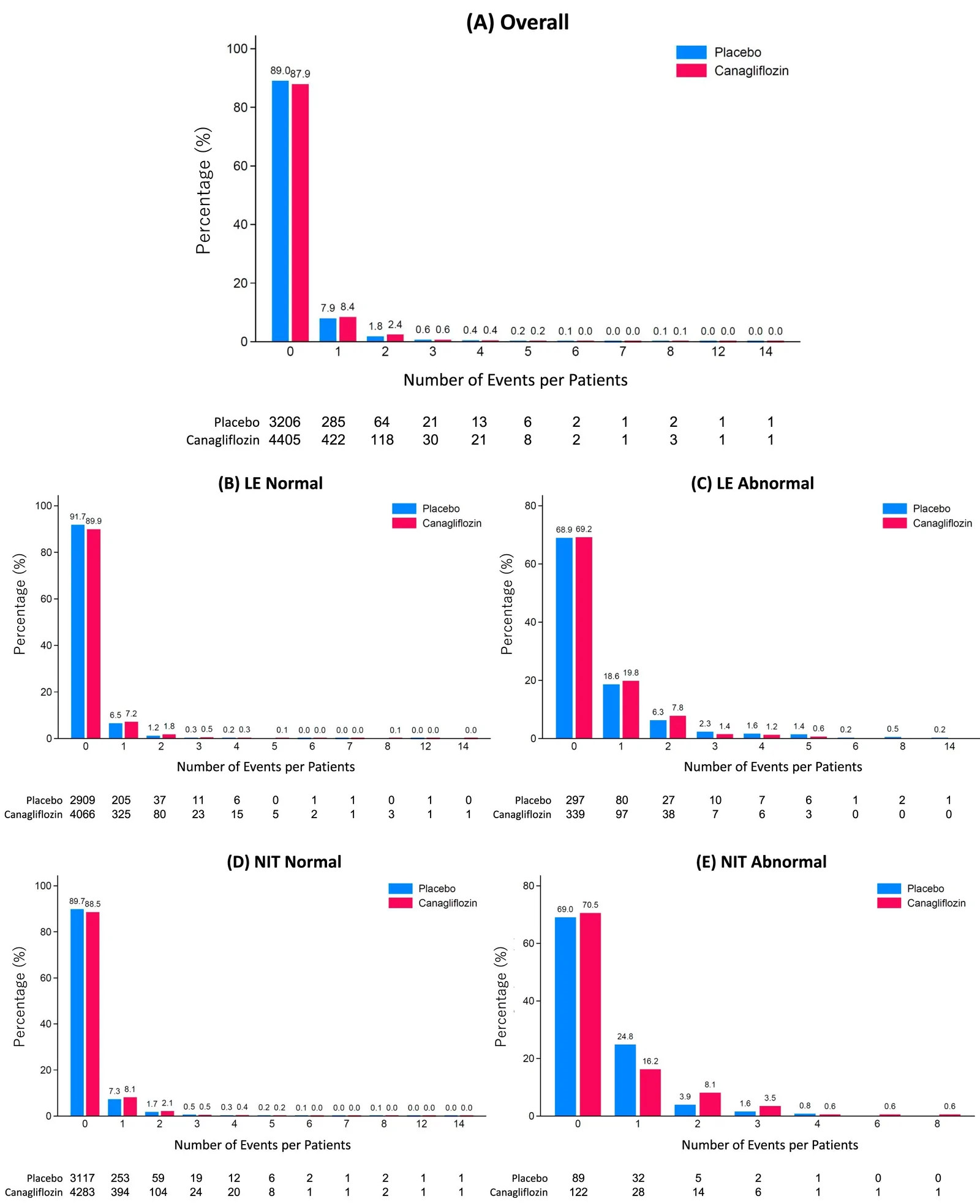
Distribution of the number of UTI episodes per patient. (**A**) Percentages of patients by number of UTI episodes during follow-up, ranging from none to a maximum of 14 events. (**B, C**) Distributions stratified by baseline LE status (normal vs abnormal), and (**D, E**) by baseline NIT status.

**Table 3:** Effects of canagliflozin versus placebo on UTI outcomes stratified by baseline dipstick results.

	Placebo		Canagliflozin			*P* interaction
	Events, *n*/*N* (%)	Event rate (per 1000 PY)	Events, *n*/*N* (%)	Event rate (per 1000 PY)	HR (95% CI)	
First UTI						
Whole	353/3602 (9.8)	41.4	550/5012 (11.0)	41.9	1.07 (0.93–1.22)	
LE test						
Three-category grouping						
Normal	237/3171 (7.5)	30.9	420/4522 (9.2)	34.8	1.17 (1.00–1.38)	.20
(1+ or 2+)	54/243 (22.2)	102.5	71/285 (24.9)	108.4	1.04 (0.73–1.49)	
(3+)	62/188 (33.0)	178.7	59/205 (28.8)	144.6	0.82 (0.57–1.17)	
Binary grouping						
Normal	237/3171 (7.5)	30.9	420/4522 (9.2)	34.8	1.17 (1.00–1.38)	.10
Abnormal	116/431 (26.9)	132.8	130/490 (26.5)	122.3	0.94 (0.73–1.21)	
NIT test						
Normal	319/3473 (9.2)	38.6	505/4839 (10.4)	39.6	1.08 (0.94–1.24)	.45
Abnormal	34/129 (26.4)	132.2	45/173 (26.0)	118.0	0.92 (0.59–1.43)	
Total (first and recurrent) UTIs						
Whole	619/3602	68.1	942/5012	67.0	1.01 (0.86–1.17)	
LE test						
Three-category grouping						
Normal	361/3171	45.1	709/4522	55.5	1.24 (1.03–1.49)	<.01
(1+ or 2+)	111/243	180.0	119/285	155.0	0.86 (0.60–1.24)	
(3+)	147/188	321.1	114/205	224.5	0.69 (0.47–0.99)	
Binary grouping						
Normal	361/3171	45.1	709/4522	55.5	1.24 (1.03–1.49)	<.01
Abnormal	258/431	240.1	233/490	182.6	0.76 (0.58–0.99)	
NIT test						
Normal	567/3473	64.6	850/4839	62.5	0.99 (0.84–1.17)	.38
Abnormal	52/129	165.0	92/173	202.3	1.18 (0.79–1.76)	

PY, person–years.

### Effect of canagliflozin on UTIs: modification by leukocyte esterase and nitrite test

Given the association between dipstick abnormalities and UTI risk, effect modification analyses were conducted using three LE categories (normal, 1–2+, 3+) and a binary classification (normal vs abnormal), while NIT was analyzed as normal vs abnormal. For the primary outcome, LE did not modify the effect of canagliflozin in either classification (*P*_interaction_ = .20 and .10), and no effect modification was observed with NIT (*P*_interaction_ = .45) (Table 3, Fig. 2). In the binary analysis, HRs for canagliflozin versus placebo were 1.17 (95% CI 1.00–1.38) in the LE-normal group and 0.94 (0.73–1.21) in the LE-abnormal group; for NIT, HRs were 1.08 (0.94–1.24) and 0.92 (0.59–1.43), respectively. For total UTI events, no heterogeneity was observed with NIT (*P*_interaction_ = .38). In contrast, LE significantly modified the treatment effect in both the two- and three-category analyses (both *P*_interaction_ < .01) (Table 3, Figs 2 and 3, Supplementary data, Fig. S5). Canagliflozin increased the risk of total UTIs in the LE-normal group (HR 1.24; 95% CI 1.03–1.49) but reduced the risk in the LE-abnormal group (HR 0.76; 95% CI 0.58–0.99). A sensitivity analysis restricted to the CREDENCE trial yielded results consistent with the primary analysis (Supplementary data, Table S5). Three-way interactions involving treatment, LE/NIT category and sex were not significant for either the primary or secondary outcome (all *P*_interaction_ > .50).

### Prediction of MGIs using leukocyte esterase and nitrite test

For MGIs, the proportional hazards assumption was violated for the treatment effect, as the hazard associated with canagliflozin was highest shortly after treatment initiation and declined over time (Supplementary data, Fig. S6). Therefore, Cox models with time-varying treatment effects were applied. In these models, neither baseline LE nor NIT predicted the risk of MGI events (Table 2, Fig. 1). Using an accelerated failure time model, canagliflozin was associated with a substantially shorter time to first MGI event compared with placebo (time ratio 0.04; 95% CI 0.02–0.07). In effect modification analyses, neither LE nor NIT altered the association between canagliflozin and MGI risk (Supplementary data, Table S6and Fig. S5).

## DISCUSSION

This study offers new insights into the associations between urine dipstick results, UTI risk and SGLT2i use in patients with T2DM. Two main findings emerged: [1] abnormal LE and NIT were associated with increased UTI risk; and [2] canagliflozin did not increase the overall risk of first or recurrent UTIs. These findings were generally consistent across both sexes.

In this study, abnormal LE and NIT results were associated with subsequent UTIs, suggesting that these findings may reflect ASB, a known precursor of symptomatic UTI in patients with T2DM [4–6, 19, 20], consistent with prior reports linking LE/NIT to ASB [21, 22]. LE and NIT are key diagnostic markers of UTI: NIT indicate bacteriuria from nitrate‐reducing organisms, while LE reflects pyuria derived from neutrophil activity [23]. We observed a clear dose–response relationship for LE but not for NIT. This difference may relate to underlying mechanisms: higher LE likely reflects stronger inflammatory activity and thus greater progression risk. Recent evidence suggests dipstick‐detected LE reflects NETosis, a neutrophil‐driven inflammatory process [24]. In contrast, NIT likely reflects bacterial burden, and a 1+ level may already represent a threshold beyond which further risk stratification becomes limited, producing a ceiling effect.

Our findings highlight the clinical utility of dipstick testing as a rapid, inexpensive and widely accessible tool for UTI risk stratification. Because of their simplicity and availability, dipstick tests are particularly useful in primary care and resource-limited settings. Identifying high-risk patients facilitates focused clinical management, potentially reducing the UTI burden [12, 25]. Additionally, dipstick markers may serve as practical criteria for identifying high-risk participants in future prevention trials.

The relationship between SGLT2i and UTIs remains a topic of ongoing debate, although several meta-analyses of randomized controlled trials and population-based cohorts have indicated no significant increase in the risk of UTIs associated with their use [13–15, 26–28]. Fralick and MacFadden suggested that the osmotic diuresis induced by SGLT2i may reduce bacterial concentration and impede bacterial ascent, potentially offsetting infection risk, although this effect may diminish over time [29]. In our study, canagliflozin did not increase the overall burden of first or recurrent UTIs over more than 3 years of follow-up. Although the underlying mechanisms are not fully understood, modest improvements in glycemic control and the influence of uromodulin may contribute to our findings. SGLT2i have been reported to increase circulating uromodulin, a renoprotective and anti-inflammatory glycoprotein produced in the thick ascending limb [30]. Increased sodium and water delivery to this segment may enhance its secretion into the tubular lumen [31]. Urinary uromodulin inhibits bacterial adhesion to the urothelium and promotes pathogen clearance [24], and higher urinary uromodulin levels have been associated with a lower risk of UTIs in prospective cohort data [32].

Surprisingly, LE status modified the treatment effect on total UTI burden: canagliflozin increased risk in patients with normal LE but reduced risk in those with abnormal LE. Although recurrent UTIs were more frequent with canagliflozin in the LE-normal group, the absolute rate difference was small (10.4 events per 1000 person-years; ∼1 excess event per 97 patient-years), suggesting limited clinical impact. In contrast, the reduction observed in the LE-abnormal group was larger (57.5 events per 1000 person-years; ∼1 prevented event per 17 patient-years). While we speculated that SGLT2i might enhance bacterial elimination by increasing uromodulin, which may act cooperatively with neutrophil extracellular traps (NETs) [24], this mechanism remains purely hypothetical. Importantly, this finding should be interpreted cautiously, as it may be due to methodological issues inherent in the study design, such as the lack of adjustment for multiple comparisons and the absence of baseline stratification by LE status.

As expected, a significant sex imbalance was observed across the LE/NIT strata. To account for the influence of sex on baseline UTI risk, we adjusted for sex in both the association and effect modification analyses. Additionally, sex-stratified analyses confirmed that the risk signals associated with abnormal dipstick findings remained consistent in direction across both sexes (Supplementary data, Table S4). Furthermore, the absence of significant three-way interactions between treatment, dipstick category and sex (all *P*_interaction_ > .50) indicates that the safety profile of canagliflozin regarding UTI risk is maintained regardless of sex. These findings suggest that the clinical utility of dipstick testing remains robust for both sexes.

In contrast, neither LE nor NIT was associated with MGI risk, and no effect modification was observed. Although LE can be positive in genital inflammation [33], dipstick testing primarily reflects urinary rather than genital conditions. Time‐to‐event analysis indicated a time‐varying treatment effect: the risk of MGI with canagliflozin was highest shortly after initiation and declined rapidly, becoming comparable to placebo over time. This transient early risk underscores the importance of early symptom monitoring and patient education on genital hygiene during treatment initiation.

A strength of this study is the use of robust data from the CANVAS and CREDENCE trials. However, several limitations should be noted. First, UTI and MGI events were investigator-reported without standardized diagnostic criteria, which may have led to inconsistent ascertainment or misclassification of events. To maintain blinding, dipstick testing was discouraged unless clinically indicated, likely resulting in capture of predominantly symptomatic rather than asymptomatic infections. Second, because only patients with T2DM were included, the findings may not generalize to individuals without diabetes. While there is no clear biological rationale for these observed associations to differ in non-diabetic populations, further studies in individuals without diabetes are warranted to confirm these results. Third, dipstick results may be affected by factors such as urine concentration and bladder retention time, influencing risk stratification accuracy. Fourth, due to the lack of multiplicity adjustments and baseline stratification by LE or nitrite status, these results should be interpreted cautiously.

In conclusion, this study indicates that urine dipstick testing has clinical value for personalized UTI risk stratification in patients with T2DM. Patients with abnormal LE and NIT results showed a higher likelihood of developing future UTIs; however, these results should not be interpreted as a reason to avoid initiating SGLT2 inhibitor therapy. Clinicians should also be cautious about attributing UTIs directly to SGLT2 inhibitor use, as unnecessary treatment changes could limit access to therapies with well-established cardiovascular and kidney benefits.

## Supplementary Material

gfag062_Supplemental_File

## Data Availability

Data from this study will be made available in the public domain via the Yale University Open Data Access Project.
