# Supplementary material for: Urinary dipstick findings and UTI risk with SGLT2 inhibitors: a post-hoc analysis of the CANVAS and CREDENCE trials
Source: Nephrol Dial Transplant. 2026 Mar 16;41(10):1887–98. doi: 10.1093/ndt/gfag062 (PMC13624796; doi:10.1093/ndt/gfag062)
Supplement: Supplementary file 1 [file gfag062_supplemental_file.docx]

**Supplementary materials**

Contents:

**Supplementary Table 1.** Summary of participant counts, person-years, and UTI event rates across the on-treatment analysis set.

**Supplementary Table 2.** Baseline characteristics according to leukocyte esterase and nitrite test in males.

**Supplementary Table 3.** Baseline characteristics according to leukocyte esterase and nitrite test in females.

**Supplementary Table 4.** Associations between baseline urine leukocyte esterase and nitrite tests and urinary tract infection incidence by sex.

**Supplementary Table 5.** Effects of canagliflozin versus placebo on UTI outcomes stratified by baseline dipstick results in CREDENCE trial

**Supplementary Table 6.** The effects of canagliflozin on mycotic genital infection according to leukocyte esterase and nitrite test.

**Supplementary Figure 1.** Flowchart of Patient Selection for Analysis Using Data from the CANVAS and CREDENCE Trials.

**Supplementary Figure 2.** Baseline distribution of LE and NIT dipstick test results in the study population (N = 8,614).

**Supplementary Figure 3.** Prevalence of abnormal LE and NIT results at baseline by age group and sex.

**Supplementary Figure 4.** Longitudinal changes in abnormal dipstick findings by treatment group.

**Supplementary Figure 5.** Impact of baseline dipstick test results and canagliflozin treatment on the risk of UTI and MGI.

**Supplementary Figure 6.** Time-varying versus time-fixed hazard ratios for the proportional hazards assumption of treatment effect.

**Supplementary Table 1.** Summary of participant counts, person-years, and UTI event rates across the on-treatment analysis set.

| **Trial** | **Treatment Arm** | **N** | **Total UTI Events** | **Person-Years** | **Event Rate (per 1000 PY)** | **Median follow-up period (25th–75th percentile), years** |
| --- | --- | --- | --- | --- | --- | --- |
| **CANVAS*** | Placebo | 1,406 | 259 | 4,185.9 | 61.9 | 4.1 (1.6–4.1) |
|  | Canagliflozin 100mg | 1,416 | 283 | 4,554.0 | 62.1 | 4.1 (2.3–4.1) |
|  | Canagliflozin 300mg | 1,396 | 292 | 4,424.4 | 66.0 |  |
| **CREDENCE**† | Placebo | 2,196 | 360 | 4,900.9 | 73.5 | 2.3 (1.8–2.9) |
|  | Canagliflozin 100mg | 2,200 | 367 | 5,077.2 | 72.3 | 2.4 (1.9–3.0) |
| **Total (pooled)** | Placebo | 3,602 | 619 | 9,086.8 | 68.1 | 2.6 (1.8–3.7) |
|  | Canagliflozin | 5,012 | 942 | 14,055.6 | 67.0 | 3.0 (2.0–4.1) |

*ClinicalTrials.gov ID: NCT01032629. Data after January 7, 2014, were excluded from the analysis for UTIs as only serious adverse events or those leading to discontinuation were recorded beyond this date. In contrast, MGIs were recorded throughout the trial regardless of severity.

†ClinicalTrials.gov ID: NCT02065791

Abbreviations: UTI, urinary tract infection; MGI, mycotic genital infections.

**Supplementary Table 2.** Baseline characteristics according to leukocyte esterase and nitrite test in males.*

|  | Leukocyte Esterase Test | | | | | Nitrite Test | | |
| --- | --- | --- | --- | --- | --- | --- | --- | --- |
|  | Normal | (1+) | (2+) | (3+) | Normal | | (1+) | (2+) |
| Characteristics | N=5,462 | N=63 | N=54 | N=120 | N=5,640 | | N=21 | N=38 |
| Age, years | 62 (9) | 64 (9) | 66 (8) | 66 (9) | 62 (9) | | 63 (9) | 67 (9) |
| Race, n (%)† |  |  |  |  |  | |  |  |
| White | 3,812 (69.8%) | 49 (77.8%) | 39 (72.2%) | 86 (71.7%) | 3,950 (70.0%) | | 14 (66.7%) | 22 (57.9%) |
| Black or African American | 166 (3.0%) | 4 (6.3%) | 1 (1.9%) | 3 (2.5%) | 172 (3.0%) | | 2 (9.5%) | 0 (0.0%) |
| Asian | 1,116 (20.4%) | 9 (14.3%) | 10 (18.5%) | 23 (19.2%) | 1,138 (20.2%) | | 5 (23.8%) | 15 (39.5%) |
| Other | 368 (6.7%) | 1 (1.6%) | 4 (7.4%) | 8 (6.7%) | 380 (6.7%) | | 0 (0.0%) | 1 (2.6%) |
| Current smoker, n (%) | 1,013 (18.5%) | 11 (17.5%) | 10 (18.5%) | 15 (12.5%) | 1,038 (18.4%) | | 4 (19.0%) | 7 (18.4%) |
| History of hypertension, n (%) | 4,964 (90.9%) | 59 (93.7%) | 54 (100.0%) | 113 (94.2%) | 5,135 (91.0%) | | 21 (100.0%) | 34 (89.5%) |
| History of CV disease, n (%) | 3,200 (58.6%) | 43 (68.3%) | 37 (68.5%) | 74 (61.7%) | 3,318 (58.8%) | | 15 (71.4%) | 21 (55.3%) |
| History of  Cerebrovascular disease, n (%) | 831 (15.2%) | 13 (20.6%) | 10 (18.5%) | 29 (24.2%) | 871 (15.4%) | | 6 (28.6%) | 6 (15.8%) |
| Diabetes duration, years | 14.4 (8.2) | 13.0 (7.3) | 16.1 (8.7) | 15.1 (8.7) | 14.4 (8.2) | | 15.1 (8.7) | 13.4 (8.0) |
| Body mass index, kg/m^2^‡ | 31 (6) | 31 (6) | 32 (6) | 31 (5) | 31 (6) | | 31 (5) | 31 (6) |
| SBP, mmHg | 138 (16) | 139 (15) | 141 (18) | 139 (16) | 138 (16) | | 137 (17) | 136 (17) |
| DBP, mmHg | 78 (10) | 76 (10) | 78 (11) | 78 (10) | 78 (10) | | 79 (13) | 74 (9) |
| Glycated hemoglobin, % | 8.1 (1.1) | 7.9 (1.1) | 8.2 (1.0) | 8.1 (1.1) | 8.1 (1.1) | | 8.5 (1.1) | 8.1 (1.2) |
| eGFR, mL/min/1.73 m^2^ | 67 (21) | 63 (23) | 65 (26) | 57 (19) | 67 (21) | | 68 (22) | 70 (21) |
| UACR, mg/g | 256 (12–953) | 371 (18–1030) | 294 (35–1143) | 522 (107–1366) | 266 (13–960) | | 400 (48–1747) | 156 (14–596) |
| Total cholesterol, mmol/L | 4.32 (1.16) | 4.42 (1.18) | 4.35 (0.80) | 4.13 (1.25) | 4.32 (1.16) | | 4.10 (1.13) | 4.30 (1.27) |
| HDL cholesterol, mmol/L | 1.11 (0.30) | 1.10 (0.34) | 1.13 (0.30) | 1.07 (0.30) | 1.11 (0.30) | | 1.01 (0.22) | 1.22 (0.51) |
| LDL cholesterol, mmol/L | 2.27 (0.94) | 2.41 (0.92) | 2.33 (0.72) | 2.18 (1.02) | 2.28 (0.93) | | 2.10 (0.97) | 2.32 (1.10) |
| Triglycerides, mmol/L | 1.73 (1.23–2.48) | 1.80 (1.19–2.39) | 1.66 (1.20–2.29) | 1.69 (1.30–2.19) | 1.73 (1.23–2.47) | | 1.93 (1.38–2.49) | 1.44 (1.16–2.57) |
| Medication, n (%) |  |  |  |  |  | |  |  |
| ACE inhibitors/ARB | 4,924 (90.2%) | 57 (90.5%) | 52 (96.3%) | 110 (91.7%) | 5,089 (90.2%) | | 20 (95.2%) | 34 (89.5%) |
| Diuretics | 2,418 (44.3%) | 25 (39.7%) | 28 (51.9%) | 46 (38.3%) | 2,489 (44.1%) | | 10 (47.6%) | 18 (47.4%) |
| Statin | 4,026 (73.7%) | 43 (68.3%) | 39 (72.2%) | 84 (70.0%) | 4,150 (73.6%) | | 15 (71.4%) | 27 (71.1%) |
| Insulin | 3,136 (57.4%) | 31 (49.2%) | 30 (55.6%) | 57 (47.5%) | 3,229 (57.3%) | | 8 (38.1%) | 17 (44.7%) |

*Data are presented as mean (interquartile range) or median (interquartile range) for continuous measures, and n (%) for categorical measures.

†Race was reported by the patients. Other includes American Indian or Alaska Native, Native Hawaiian or other Pacific Islander, multiple, other, unknown, and not reported.

‡The body-mass index is the weight in kilograms divided by the square of the height in meters

Abbreviations: CV, cardiovascular; SBP, systolic blood pressure; DBP, diastolic blood pressure; eGFR, estimated glomerular filtration rate; UACR, urinary albumin-to-creatinine ratio; HDL, high–density lipoprotein; LDL, low–density lipoprotein; ACE, angiotensin–converting enzyme; ARB, angiotensin receptor blockers.

**Supplementary Table 3.** Baseline characteristics according to leukocyte esterase and nitrite test in females.*

|  | Leukocyte Esterase Test | | | | | Nitrite Test | | |
| --- | --- | --- | --- | --- | --- | --- | --- | --- |
|  | Normal | (1+) | (2+) | (3+) | Normal | | (1+) | (2+) |
| Characteristics | N=2,231 | N=222 | N=189 | N=273 | N=2,672 | | N=70 | N=173 |
| Age, years | 62 (9) | 63 (8) | 63 (8) | 63 (9) | 62 (9) | | 62 (8) | 63 (8) |
| Race, n (%)† |  |  |  |  |  | |  |  |
| White | 1,573 (70.5%) | 154 (69.4%) | 123 (65.1%) | 179 (65.6%) | 1,857 (69.5%) | | 53 (75.7%) | 119 (68.8%) |
| Black or African American | 116 (5.2%) | 13 (5.9%) | 12 (6.3%) | 13 (4.8%) | 145 (5.4%) | | 2 (2.9%) | 7 (4.0%) |
| Asian | 369 (16.5%) | 41 (18.5%) | 36 (19.0%) | 51 (18.7%) | 453 (17.0%) | | 9 (12.9%) | 35 (20.2%) |
| Other | 173 (7.8%) | 14 (6.3%) | 18 (9.5%) | 30 (11.0%) | 217 (8.1%) | | 6 (8.6%) | 12 (6.9%) |
| Current smoker, n (%) | 276 (12.4%) | 24 (10.8%) | 16 (8.5%) | 25 (9.2%) | 315 (11.8%) | | 4 (5.7%) | 22 (12.7%) |
| History of hypertension, n (%) | 2,110 (94.6%) | 213 (95.9%) | 178 (94.2%) | 255 (93.4%) | 2,528 (94.6%) | | 67 (95.7%) | 161 (93.1%) |
| History of CV disease, n (%) | 1,047 (46.9%) | 86 (38.7%) | 81 (42.9%) | 132 (48.4%) | 1,238 (46.3%) | | 33 (47.1%) | 75 (43.4%) |
| History of  Cerebrovascular disease, n (%) | 396 (17.7%) | 29 (13.1%) | 36 (19.0%) | 43 (15.8%) | 465 (17.4%) | | 10 (14.3%) | 29 (16.8%) |
| Diabetes duration, years | 14.8 (8.1) | 15.5 (8.2) | 14.3 (7.7) | 16.2 (8.6) | 15.0 (8.2) | | 14.5 (7.9) | 14.3 (8.3) |
| Body mass index, kg/m^2^‡ | 32 (7) | 33 (6) | 32 (6) | 33 (7) | 32 (7) | | 33 (7) | 33 (7) |
| SBP, mmHg | 139 (16) | 139 (16) | 140 (16) | 138 (17) | 139 (16) | | 141 (17) | 140 (16) |
| DBP, mmHg | 78 (9) | 77 (9) | 77 (9) | 76 (9) | 78 (9) | | 78 (10) | 78 (8) |
| Glycated hemoglobin, % | 8.4 (1.2) | 8.3 (1.1) | 8.3 (1.2) | 8.4 (1.2) | 8.4 (1.2) | | 8.5 (1.2) | 8.4 (1.2) |
| eGFR, mL/min/1.73 m^2^ | 67 (21) | 62 (21) | 62 (21) | 61 (21) | 66 (21) | | 66 (20) | 66 (21) |
| UACR, mg/g | 214 (11–1054) | 231 (16–1125) | 240 (14–1045) | 268 (21–1139) | 232 (11–1087) | | 238 (19–1007) | 115 (18–852) |
| Total cholesterol, mmol/L | 4.89 (1.33) | 4.93 (1.37) | 4.81 (1.27) | 4.73 (1.25) | 4.87 (1.31) | | 5.08 (1.66) | 4.83 (1.36) |
| HDL cholesterol, mmol/L | 1.29 (0.36) | 1.27 (0.32) | 1.29 (0.37) | 1.26 (0.35) | 1.29 (0.36) | | 1.21 (0.34) | 1.29 (0.34) |
| LDL cholesterol, mmol/L | 2.64 (1.09) | 2.62 (1.10) | 2.57 (1.05) | 2.55 (1.07) | 2.62 (1.08) | | 2.71 (1.16) | 2.66 (1.14) |
| Triglycerides, mmol/L | 1.76 (1.29–2.47) | 1.75 (1.34–2.62) | 1.74 (1.37–2.54) | 1.73 (1.27–2.40) | 1.76 (1.30–2.49) | | 1.73 (1.15–2.59) | 1.63 (1.24–2.26) |
| Medication, n (%) |  |  |  |  |  | |  |  |
| ACE inhibitors/ARB | 2,015 (90.3%) | 203 (91.4%) | 177 (93.7%) | 254 (93.0%) | 2,420 (90.6%) | | 66 (94.3%) | 163 (94.2%) |
| Diuretics | 1,042 (46.7%) | 117 (52.7%) | 91 (48.1%) | 139 (50.9%) | 1,269 (47.5%) | | 34 (48.6%) | 86 (49.7%) |
| Statin | 1,453 (65.1%) | 154 (69.4%) | 121 (64.0%) | 173 (63.4%) | 1,750 (65.5%) | | 40 (57.1%) | 111 (64.2%) |
| Insulin | 1,296 (58.1%) | 148 (66.7%) | 117 (61.9%) | 183 (67.0%) | 1,588 (59.4%) | | 44 (62.9%) | 112 (64.7%) |

*Data are presented as mean (interquartile range) or median (interquartile range) for continuous measures, and n (%) for categorical measures.

†Race was reported by the patients. Other includes American Indian or Alaska Native, Native Hawaiian or other Pacific Islander, multiple, other, unknown, and not reported.

‡The body-mass index is the weight in kilograms divided by the square of the height in meters

Abbreviations: CV, cardiovascular; SBP, systolic blood pressure; DBP, diastolic blood pressure; eGFR, estimated glomerular filtration rate; UACR, urinary albumin-to-creatinine ratio; HDL, high–density lipoprotein; LDL, low–density lipoprotein; ACE, angiotensin–converting enzyme; ARB, angiotensin receptor blockers.

**Supplementary Table 4.** Associations between baseline urine leukocyte esterase and nitrite tests and urinary tract infection incidence by sex.

|  | Events, n/N (%) | Event Rate  (per 1000 PY) | Model 3*  HR (95% CI) | Events, n/N (%) | Event Rate  (per 1000 PY) | Model 3*  HR (95% CI) | P interaction† |
| --- | --- | --- | --- | --- | --- | --- | --- |
|  | **Male** | | | **Female** | | |  |
| Whole | 342/5699 (6.0) | 23.0 |  | 561/2915 (19.3) | 82.5 |  |  |
| **LE Test** |  |  |  |  |  |  |  |
| Normal | 296/5462 (5.4) | 20.6 | ref | 361/2231 (16.2) | 67.3 | ref | <0.01 |
| (1+) | 11/63 (17.5) | 79.3 | 3.21 (1.75–5.88) | 53/222 (23.9) | 108.0 | 1.50 (1.12–2.01) |  |
| (2+) | 8/54 (14.8) | 62.3 | 2.83 (1.40–5.74) | 53/189 (28.0) | 125.1 | 1.74 (1.30–2.32) |  |
| (3+) | 27/120 (22.5) | 113.1 | 4.56 (3.04–6.86) | 94/273 (34.4) | 182.1 | 2.41 (1.91–3.03) |  |
| **NIT Test** |  |  |  |  |  |  |  |
| Normal | 333/5640 (5.9) | 22.6 | ref | 491/2672 (18.4) | 78.0 | ref | 0.30 |
| (1+) | 3/21 (14.3) | 63.1 | 2.43 (0.84–8.28) | 25/70 (35.7) | 178.4 | 2.24 (1.50–3.35) |  |
| (2+) | 6/38 (15.8) | 69.6 | 2.77 (1.23–6.25) | 45/173 (26.0) | 123.4 | 1.58 (1.16–2.14) |  |

*Model 3: adjusted for age, sex, treatment (canagliflozin vs. placebo), race, history of cerebrovascular disease, diabetes duration, body mass index, glycated hemoglobin, eGFR, UACR, diuretic use, and insulin use.

†P-values represent interactions between categories of leukocyte esterase or nitrite tests and sex.

Abbreviations: PY, person-years; HR, hazard ratio; CI, confidence interval; LE, leukocyte esterase; NIT, nitrite; eGFR, estimated glomerular filtration rate; UACR, urinary albumin-to-creatinine ratio

**Supplementary Table 5.** Effects of canagliflozin versus placebo on UTI outcomes stratified by baseline dipstick results in CREDENCE trial

|  | Placebo | | Canagliflozin | |  | P interaction |
| --- | --- | --- | --- | --- | --- | --- |
|  | Events, n/N (%) | Event rate  (per 1000 PY) | Events, n/N (%) | Event rate  (per 1000 PY) | HR (95% CI) |  |
| First Urinary Tract Infection | | | | | | |
| Whole | 221/2196 (10.1) | 47.9 | 245/2200 (11.1) | 51.3 | 1.07 (0.89–1.28) |  |
| LE Test |  |  |  |  |  |  |
| Three-category grouping | |  |  |  |  |  |
| Normal | 142/1915 (7.4) | 34.7 | 168/1949 (8.6) | 39.0 | 1.12 (0.90–1.41) | 0.75 |
| (1+ or 2+) | 35/151 (23.2) | 119.6 | 40/140 (28.6) | 147.2 | 1.19 (0.75–1.88) |  |
| (3+) | 44/130 (33.8) | 197.9 | 37/111 (33.3) | 191.4 | 0.94 (0.61–1.45) |  |
| Binary grouping |  |  |  |  |  |  |
| Normal | 142/1915 (7.4) | 34.7 | 168/1949 (8.6) | 39.0 | 1.12 (0.90–1.41) | 0.67 |
| Abnormal | 79/281 (28.1) | 153.4 | 77/251 (30.7) | 165.5 | 1.07 (0.78–1.46) |  |
| NIT Test |  |  |  |  |  |  |
| Normal | 200/2117 (9.4) | 44.8 | 222/2125 (10.4) | 47.9 | 1.07 (0.88–1.29) | 0.97 |
| Abnormal | 21/79 (26.6) | 140.5 | 23/75 (30.7) | 163.1 | 1.14 (0.63–2.06) |  |
| Total (first and recurrent) Urinary Tract Infections | | | | | | |
| Whole | 360/2196 | 73.5 | 367/2200 | 72.3 | 0.97 (0.79–1.20) |  |
| LE Test |  |  |  |  |  |  |
| Three-category grouping | |  |  |  |  |  |
| Normal | 194/1915 | 45.5 | 247/1949 | 54.7 | 1.18 (0.92–1.53) | 0.17 |
| (1+ or 2+) | 64/151 | 185.4 | 61/140 | 189.1 | 1.02 (0.67–1.56) |  |
| (3+) | 102/130 | 350.7 | 59/111 | 251.1 | 0.69 (0.43–1.11) |  |
| Binary grouping |  |  |  |  |  |  |
| Normal | 194/1915 | 45.5 | 247/1949 | 54.7 | 1.18 (0.92–1.53) | 0.06 |
| Abnormal | 166/281 | 261.0 | 120/251 | 215.2 | 0.82 (0.58–1.15) |  |
| NIT Test |  |  |  |  |  |  |
| Normal | 332/2117 | 70.3 | 327/2125 | 66.6 | 0.94 (0.75–1.17) | 0.18 |
| Abnormal | 28/79 | 154.8 | 40/75 | 235.0 | 1.43 (0.85–2.41) |  |

Abbreviations: PY, person–years; HR, hazard ratio; CI, confidence interval; LE, leukocyte esterase; NIT, nitrite

**Supplementary Table 6.** The effects of canagliflozin on mycotic genital infection according to leukocyte esterase and nitrite test.

|  | Placebo | | Canagliflozin | | |  | P interaction |
| --- | --- | --- | --- | --- | --- | --- | --- |
|  | Events, n/N (%) | Event rate  (per 1000 PY) | Events, n/N (%) | Event rate  (per 1000 PY) | Time ratio (95% CI) | |  |
| Whole | 65/3602 (1.8) | 6.5 | 459/5012 (9.2) | 30.2 | 0.04 (0.02–0.07) | |  |
| LE Test |  |  |  |  |  | |  |
| Three-category grouping | |  |  |  |  | |  |
| Normal | 54/3171 (1.7) | 6.1 | 407/4522 (9.0) | 29.1 | 0.04 (0.02–0.06) | | 0.41 |
| (1+ or 2+) | 5/243 (2.1) | 7.9 | 33/285 (11.6) | 45.4 | 0.03 (0.00–0.18) | |  |
| (3+) | 6/188 (3.2) | 13.0 | 19/205 (9.3) | 39.1 | 0.13 (0.02–0.82) | |  |
| Binary grouping | |  |  |  |  | |  |
| Normal | 54/3171 (1.7) | 6.1 | 407/4522 (9.0) | 29.1 | 0.04 (0.02–0.06) | | 0.66 |
| Abnormal | 11/431 (2.6) | 10.1 | 52/490 (10.6) | 42.9 | 0.06 (0.02–0.21) | |  |
| NIT Test |  |  |  |  |  | |  |
| Normal | 63/3473 (1.8) | 6.5 | 440/4839 (9.1) | 29.8 | 0.04 (0.02–0.07) | | 0.57 |
| Abnormal | 2/129 (1.6) | 6.3 | 19/173 (11.0) | 44.1 | 0.01 (0.00–0.26) | |  |

Since the Cox proportional hazards assumption was violated, time ratios were estimated using Accelerated Failure Time models adjusted for age and sex. The Log-Normal distribution was chosen as the best fit for these models based on AIC, BIC, and Cox-Snell residuals.

Abbreviations: PY, person-years; CI, confidence interval; LE, leukocyte esterase; NIT, nitrite; AIC, Akaike Information Criterion; BIC, Bayesian Information Criterion

**Supplementary Figure 1.** Flowchart of Patient Selection for Analysis Using Data from the CANVAS and CREDENCE Trials.

**Supplementary Figure 2.** Baseline distribution of LE and NIT dipstick test results in the study population (N = 8,614).

This Venn diagram illustrates the overlap and exclusivity of abnormal LE and NIT test results. Among the total population, 7,560 individuals (87.8%) had normal results for both tests; 752 (8.7%) had isolated abnormal LE; 133 (1.5%) had isolated abnormal NIT; and 169 (2.0%) had abnormalities in both tests.

Abbreviations: LE, leukocyte esterase; NIT, nitrite.

**Supplementary Figure 3.** Prevalence of abnormal LE and NIT results at baseline by age group and sex.

Panel A shows the prevalence of abnormal LE results stratified by age group and sex, presented using a stacked bar chart with three levels of abnormality (1+, 2+, 3+). Panel B presents the corresponding distribution of NIT results, which include two levels of abnormality (1+, 2+).

Abbreviations: LE, leukocyte esterase; NIT, nitrite.

**Supplementary Figure 4.** Longitudinal changes in abnormal dipstick findings by treatment group.

Panel A shows changes in the proportions of patients with abnormal LE results (1+, 2+, 3+) over time, stratified by treatment group (canagliflozin vs. placebo). Panel B shows corresponding changes for abnormal NIT results (1+, 2+). Stacked bar charts illustrate yearly trends over a three-year follow-up period.

Abbreviations: LE, leukocyte esterase; NIT, nitrite.

**Supplementary Figure 5.** Impact of baseline dipstick test results and canagliflozin treatment on the risk of UTI and MGI.

Panel A shows Kaplan–Meier plots for time to first UTI according to baseline LE categories (normal, 1+ or 2+, 3+). Panel B shows cumulative incidence plots for total UTI events based on the same LE categories. Panels C and D present cumulative incidence plots for time to first MGI by LE category, using both ordinal (normal, 1+ or 2+, 3+) and binary (normal vs. abnormal) classifications. Panel E shows Kaplan–Meier plots for time to first MGI by treatment group according to NIT status (normal vs. abnormal).

Abbreviations: UTI, urinary tract infection; MGI, mycotic genital infection; LE, leukocyte esterase; NIT, nitrite.

**Supplementary Figure 6.** Time-varying versus time-fixed hazard ratios for the proportional hazards assumption of treatment effect.

Panels A and B depict time-varying versus time-fixed hazard ratios for the effect of canagliflozin versus placebo on UTI and MGI, respectively. The analysis was conducted using the “stphcoxrcs” command in Stata, which models the interaction between treatment and log-transformed time using restricted cubic splines. Dashed lines represent 95% confidence intervals. The proportional hazards assumption was satisfied for UTI (A) but violated for MGI (B).

Abbreviations: UTI, urinary tract infection; MGI, mycotic genital infection.
